# Therapeutic Utility of Mulligan Traction Straight Leg Raise Stretch and Proprioceptive Exercises in Osteoarthritis Treatment

**DOI:** 10.7759/cureus.74382

**Published:** 2024-11-25

**Authors:** Saman Iqbal, Imran Ahmad Khan, Maliha Khalid Khan, Qurat-Ul- Ain, Hifza Arif, Komal Ammar Bukhari, Rukhsana Bibi, Urwa Batool, Mahnoor Shahzad, Tehreem Saad

**Affiliations:** 1 Ali-Ul-Murtaza, Department of Rehabilitation Sciences, Muhammad Institute of Medical and Allied Sciences, Multan, PAK; 2 Department of Pharmacy, Muhammad Nawaz Sharif University of Agriculture, Multan, PAK; 3 Department of Pathobiology and Biomedical Sciences, Muhammad Nawaz Sharif University of Agriculture, Multan, PAK

**Keywords:** functional activities, mulligan traction, osteoarthritis, physical therapy, proprioceptive exercises

## Abstract

Background

Given the increasing incidence and severity of knee osteoarthritis (OA), it is crucial to investigate and refine therapeutic approaches.

Aim

The objective of this study is to evaluate the effectiveness and potential synergistic effects of proprioceptive exercises combined with Mulligan traction straight leg raise (MT-SLR) in treating OA. This includes improving symptoms such as functional mobility, pain reduction, and relevant serological markers, highlighting the potential of this approach to enhance overall patient outcomes.

Methods

A pragmatic, community-based, parallel-group, double-blinded, randomized clinical trial was conducted from August 2023 to March 2024. A probability convenience sampling technique was used to calculate the sample size. A total of 130 pre-diagnosed OA patients were randomly assigned to three groups via the lottery method. Group A received proprioceptive exercises, group B received the MT-SLR, and group C received both proprioceptive exercises and MT-SLR. The Pain Catastrophizing Scale (PCS), Numerical Pain Rating Scale, Western Ontario and McMaster University Osteoarthritis Index (WOMAC), erythrocyte sedimentation rate (ESR), flexion, CRP, bone electrolytes in blood (calcium, magnesium, and phosphorus), and X-rays were used to quantify pain, stiffness, functional activities, biochemical parameters, and radiographic findings at baseline and after the 24th session. Data were analyzed using IBM SPSS Statistics for Windows, Version 23.0 (Released 2015; IBM Corp., Armonk, NY, USA), with a p-value of <0.05 considered statistically significant.

Results

Out of the 130 participants, 126 were analyzed at the 24th session, comprising 50 males and 76 females. After 24 sessions, Group C (combined therapy) exhibited the most significant improvements across all parameters (p = 0.000), including flexion range of motion, WOMAC Pain, activities of daily living, stiffness, and PCS, outperforming both Group A (p = 0.011) and Group B (p = 0.005). Group C also demonstrated the greatest reductions in ESR (p < 0.001), CRP (p < 0.001), and IL-6 (p < 0.001), along with the highest improvements in calcium, magnesium, and phosphorus levels (p < 0.005). X-ray assessments revealed that Group C had the most notable joint space recovery, while Group B showed no changes.

Conclusions

In the treatment of knee OA, proprioceptive exercises have been found to be more effective than the MT-SLR stretch. Given the rising prevalence and severity of knee OA, this study emphasizes the need to explore and refine treatment strategies. The findings indicate that combining MT-SLR with proprioceptive exercises yields superior results compared to either intervention alone. The group receiving the combined therapy showed the most significant improvements across several key measures, including functional mobility, pain reduction, and relevant serological markers, highlighting the potential of this approach to enhance overall patient outcomes. The notable differences in performance between the treatment groups suggest that a more comprehensive, integrative approach to knee OA could lead to better management outcomes.

## Introduction

Millions of people worldwide suffer from osteoarthritis (OA), a common and debilitating degenerative joint condition, particularly among the elderly [1]. OA is characterized by the progressive loss of articular cartilage, leading to pain, stiffness, reduced range of motion (ROM), and significant functional limitations. These symptoms not only restrict movement but are also major contributors to impairment, substantially reducing the quality of life for affected individuals [2]. Primary OA is characterized by cartilage degradation with no known etiology, while secondary OA arises due to abnormal cartilage wear or decreased force absorption, often following joint injury. Key risk factors for knee OA include obesity, gender, previous knee injuries, aging, and genetic predisposition [3]. Chronic synovial expansion, a hallmark of knee OA, exacerbates the progression of the disease and its symptoms [4].

In the cartilage and subchondral bone, chondrocytes and osteoblasts are exposed to a variety of inflammatory mediators and receptors [3]. The pathogenesis of OA is heavily influenced by immune cells and their cytokines, such as TNF-α, IL-1β, and IL-6, which play a crucial role in tissue damage, cell destruction, and the inflammatory response [4]. While conservative treatments remain the first-line approach for most patients, advanced cases may require surgical intervention, such as total knee replacement. Physiotherapy plays a crucial role in managing knee OA, offering therapies aimed at improving function, reducing pain, and increasing mobility [5]. Among the various physiotherapy options, Mulligan traction straight leg raise (MT-SLR) stretch and proprioceptive exercises are widely utilized in the conservative management of knee OA. However, each method operates through different mechanisms, necessitating further investigation of their relative efficacy [6].

The Mulligan concept, which emphasizes mobilization with movement, is the basis for the MT-SLR stretch [6]. This technique seeks to improve joint alignment and reduce discomfort by gently applying traction to the knee joint during a straight leg raise. MT-SLR can help reduce joint compression, leading to less pain and an improved ROM in patients with knee OA [7].

Proprioception refers to the ability to sense joint position and movement, which relies on mechanoreceptors. The sensitivity of proprioception is determined by the number of these mechanoreceptors [8]. Proprioceptive exercises aim to enhance the body’s awareness of joint position and movement, often compromised in individuals with OA [9]. These exercises improve joint stability, balance, and coordination, which are essential for reducing the risk of falls and improving functional outcomes. Given that proprioceptive deficits can exacerbate pain and mobility issues in knee OA, proprioceptive exercises are a key component of treatment [9].

Although both MT-SLR and proprioceptive exercises are commonly used in clinical settings to manage knee OA, there is limited direct comparison of their respective efficacy in the literature. This gap underscores the need for further research comparing these two interventions. The hypothesis of this study is that MT-SLR will offer more benefits than proprioceptive exercises in terms of pain relief, functional improvement, and ROM for patients with knee OA.

The objective of this study is to assess and compare the effectiveness of proprioceptive exercises and MT-SLR in the conservative treatment of knee OA. Specifically, the study aims to evaluate the impact of these therapies on pain management, functional enhancement, and ROM in individuals with knee OA. By providing evidence-based insights, this study seeks to bridge the gap in existing literature and inform clinical decision-making, ultimately improving treatment outcomes for patients with knee OA.

## Materials and methods

This pragmatic, community-based, parallel-group, double-blinded, randomized clinical trial was conducted at the Muhammad Physical Therapy Clinic and Rehabilitation Center in Multan, Pakistan, from August 2023 to March 2024. The trial was approved by the Registry of Clinical Trials (IRCT20230202057310N2), and the statistical analyzer and outcome assessor were blinded to minimize bias.

Following approval from the Institutional Ethical Committee (MIMAS/06/26/Maliha), the clinical trial was registered, and the study adhered to the Helsinki Declaration for Human Research (2013) throughout the procedure [10].

The sample size for the study was calculated using G*Power software (version 3.1.9.7), assuming a medium effect size (f = 0.25), three groups, and a significance level (α) of 0.05. A statistical power (1 - β) of 0.80 was targeted to detect clinically significant differences between the groups. Based on this calculation, the initial sample size was determined. To compensate for potential participant dropout, the sample size was increased by 10%, which is standard practice in randomized controlled trials to maintain the reliability of the results [11].

An orthopedic surgeon pre-diagnosed 130 patients with OA, who were then randomly assigned to Groups A, B, and C. A qualified research assistant, who was not involved in the patients’ clinical care, performed the randomization and allocation, ensuring a blinded procedure. Random sampling was employed to select participants, and the lottery method was used for random allocation. Prior to recruitment, all participants were provided with comprehensive information about the study, including its objectives, methods, potential risks, and benefits. Informed consent was obtained from all participants, confirming their voluntary participation.

Eligible participants were aged 40-65 years, of either gender, with cartilage degradation of grade II or higher, local biomechanical factors (such as joint deformity and muscle weakness), nutritional influences, and postmenopausal status. Kellgren-Lawrence grading was determined through radiography [12].

Exclusion criteria included patients with knee replacements, neuropathy, rheumatoid arthritis, knee ligament injuries, neurological conditions, knee fractures, meniscus injuries, diabetes mellitus for over eight years, and metal knee implants. OA was assessed in all participants using physical, serological, and radiological examinations (X-rays).

The proprioceptive activities assigned to Group A included half squats, standing on one foot, walking on heels and toes (both with eyes open and closed), one-legged balance exercises with a heating pad, and knee flexion and extension exercises performed while seated. Group A participants completed these exercises three times a week for eight weeks, totaling 24 sessions. Each session lasted 45 minutes. The exercises began with low-to-moderate intensity balance and stability drills, such as single-leg stances and balance exercises on unstable surfaces, progressing from static to dynamic exercises. During one-legged balancing exercises, participants used a heating pad on the affected knee for 10-15 minutes to promote relaxation and improve blood circulation to the joint before starting the exercises [13].

Each exercise consisted of two to three sets of 10-15 repetitions, with each set lasting one to two minutes and a 30-second rest between sets. Intensity was adjusted as necessary based on participant progress to ensure the exercises remained appropriately challenging [13]. Group B, receiving MT-SLR stretch, performed the intervention three times a week for 30 minutes per session. Each session involved three sets of 10 repetitions of straight leg lifts while applying manual traction, with a 30-second break between sets. The traction force was tailored to each participant's comfort level, with repetitions held for 15-20 seconds, followed by a 30-second rest between sets [14]. Group C received both proprioceptive exercises and MT-SLR.

All groups were supervised by a senior physiotherapist and a senior medical doctor to ensure proper technique and progress. Pain and functionality were assessed using the Western Ontario and McMaster University Osteoarthritis Index (WOMAC), which is known for its excellent test-retest reliability and strong construct validity [15]. Pain catastrophizing was measured using the Pain Catastrophizing Scale (PCS), which has high internal consistency and good test-retest reliability [16]. Pain was also assessed using the Numerical Pain Rating Scale (NPRS), which has excellent test-retest reliability and strong internal consistency, with good concurrent validity against other pain measures [17]. Erythrocyte sedimentation rate (ESR), CRP, IL-6, bone electrolytes (calcium, magnesium, and phosphorus), and X-rays were used to assess pain, stiffness, functional activities, biochemical, and radiological parameters at baseline and the 24th session.

Quantitative data were analyzed using IBM SPSS Statistics for Windows, Version 23.0 (Released 2015; IBM Corp., Armonk, NY, USA). Statistical significance was determined with a p-value of less than 0.05. The normality of the data was assessed using the Shapiro-Wilk test before conducting statistical analyses. Based on the results of the normality test, parametric tests were applied to normally distributed data. A paired t-test was used to assess differences within groups, and one-way ANOVA was used to compare differences across groups.

## Results

**Figure 1:**
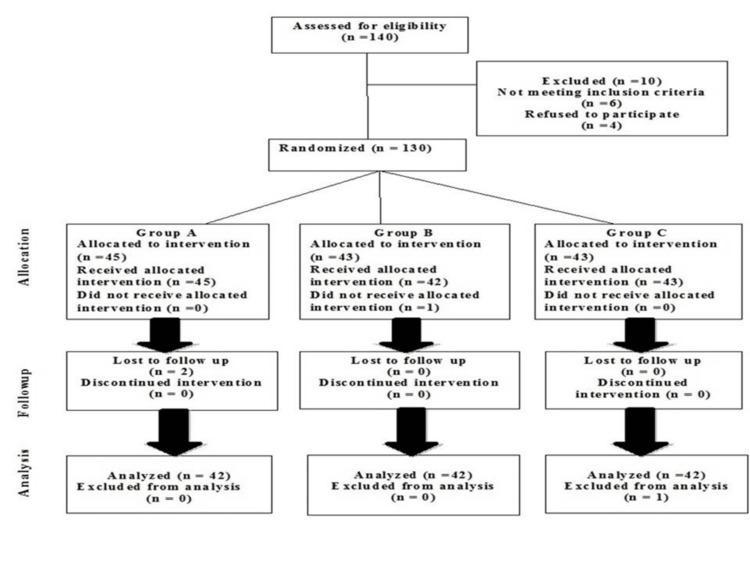
CONSORT diagram illustrating the flow of participants through each stage of the randomized trial

Out of the 130 participants, 126 were analyzed at the 24th session, while four participants were lost to follow-up (Figure 1). The cohort consisted of 50 males (39.68%) and 76 females (60.32%). The demographic data of the participants are presented in Table 1.

**Table 1:** Demographic data of participants K-L grades: Kellgren-Lawrence; OA: osteoarthritis

Variables	Group A, n (%)	Group B, n (%)	Group C, n (%)	Total, N (%)
Gender				
Male	19 (45.2)	16 (38.09)	15 (35.71)	50 (39.68)
Female	23 (54.76)	26 (61.90)	27 (64.28)	76 (60.32)
K-L grades of OA				
K-L Grade 1	14 (31.11)	13 (30.95)	16 (37.09)	43 (33.07)
K-L Grade 2	15 (33.33)	15 (31.11)	16 (37.09)	46 (35.38)
K-L Grade 3	14 (31.11)	10 (22.22)	7 (16.27.33)	43 (23.84)
K-L Grade 4	2 (4.44)	4 (8.88)	4 (9.30)	10 (7.69)

A total of 126 patients were assessed for flexion, PCS, NPRS, WOMAC, and biochemical parameters (ESR, CRP, and IL-6). In contrast, Group B was also evaluated using the same parameters (flexion, PCS, NPRS, WOMAC, and biochemical markers), but the results were less effective. The mean and SD for baseline measurements and the 24th session are presented for all outcomes in Table 2, Table 3, and Table 4.

**Table 2:** Comparison of flexion, WOMAC Pain, WOMAC ADLs, WOMAC Stiffness, NPRS, and PCS scores at baseline and after 24 sessions in Group A (proprioceptive exercises), Group B (Mulligan traction), and Group C (combined therapy) Data are presented as mean ± SD with corresponding within-group and between-group p-values. ADLs: activities of daily living; NPRS: Numerical Pain Rating Scale; PCS: Pain Catastrophizing Scale; ROM: range of motion; WOMAC: Western Ontario and McMaster University Arthritis Index

Parameters	Time point	Group A	Within Group A (p-value)	Group B	Within Group B (p-value)	Group C	Within Group C (p-value)	Between groups
Flexion (ROM)	Baseline	84.6 ± 8.86	0.011	84.6 ± 8.86	0.005	84.6 ± 8.86	0.000	0.000
	24th session	91.97 ± 14.55		114.42 ± 6.96		84.6 ± 8.86		
WOMAC Pain	Baseline	87.42 ± 6.63	0.005	87.42 ± 6.63	0.044	87.00 ± 5.85	0.000	0.000
	24th session	23.76 ± 6.63		78.33 ± 6.59		16.2 ± 7.45		
WOMAC ADLs	Baseline	57.66 ± 20.17	0.004	57.66 ± 20.17	0.007	57.33 ± 12.99	0.001	0.000
	24th session	29.56 ± 16.47		39.56 ± 16.47		12.69 ± 8.91		
WOMAC Stiffness	Baseline	7.09 ± 1.03	0.005	7.90 ± 1.17	0.082	7.09 ± 1.03	0.002	0.001
	24th session	2.84 ± 1.77		4.70 ± 0.62		2.84 ± 1.77		
NPRS	Baseline	7.58 ± 0.70	0.002	7.29 ± 1.28	0.004	7.48 ± 0.70	0.000	0.000
	24th session	3.98 ± 0.71		4.54 ± 1.29		1.98 ± 0.71		
PCS	Baseline	33.61 ± 4.38	0.001	33.61 ± 4.93	0.005	36.61 ± 4.93	0.000	0.000
	24th session	21.61 ± 3.78		28.61 ± 3.72		15.61 ± 3.72		

**Table 3:** Comparison of ESR, CRP, and IL-6 levels at baseline and after 24 sessions in Group A (proprioceptive exercises), Group B (Mulligan traction), and Group C (combined therapy) Data are presented as mean ± SD with corresponding within-group and between-group p-values. ESR: erythrocyte sedimentation rate

Parameters	Time point	Group A	Within Group A (p-value)	Group B	Within Group B (p-value)	Group C	Within Group C (p-value)	Between groups
ESR	Baseline	91 ± 20.9	0.002	91 ± 20.9	0.005	92 ± 22.9	0.001	0.000
	24th session	59 ± 6.1		79 ± 6.1		1.1 ± 0.7		
CRP	Baseline	10.1 ± 7	0.002	10.1 ± 7	0.006	11.2 ± 6.1	0.001	0.000
	24th session	5 ± 3.4		8 ± 3.4		6.31 ± 3.0		
IL-6	Baseline	137 ± 35	0.007	138 ± 36	0.005	141 ± 38	0.001	0.001
	24th session	104 ± 12		84 ± 12		64 ± 12		

**Table 4:** Comparison of calcium, phosphorous, and magnesium levels at baseline and after 24 sessions in Group A (proprioceptive exercises), Group B (Mulligan traction), and Group C (combined therapy) Data are presented as mean ± SD with corresponding within-group and between-group p-values.

Parameters	Time point	Group A	Within Group A (p-value)	Group B	Within Group B (p-value)	Group C	Within Group C (p-value)	Between groups
Calcium (mg/dL)	Baseline	3.63 ± 0.04	0.007	3.43 ± 0.01	0.005	3.63 ± 0.04	0.001	0.000
	24th session	5.43 ± 0.05		7.68 ± 0.01		9.43 ± 0.05		
Magnesium (mg/dL)	Baseline	1.8 ± 0.21	0.007	1.9 ± 0.22	0.005	1.7 ± 0.01	0.002	0.001
	24th session	2.00 ± 0.02		2.10 ± 0.04		2.26 ± 0.02		
Phosphorous (mg/dL)	Baseline	3.05 ± 0.13	0.005	3.38 ± 0.12	0.003	3.48 ± 0.32	0.002	0.001
	24th session	3.95 ± 0.03		4.01 ± 0.52		4.02 ± 0.52		

Flexion ROM, WOMAC Pain, WOMAC activities of daily living (ADLs), WOMAC Stiffness, NPRS, and PCS showed no significant differences between the three groups at baseline. However, after 24 sessions, significant improvements were observed in Group A (proprioceptive exercises) compared to baseline, with flexion ROM, WOMAC Pain, WOMAC ADLs, WOMAC Stiffness, NPRS, and PCS showing notable improvements (p = 0.011). In Group B (MT-SLR), flexion ROM, WOMAC Pain, WOMAC ADLs, NPRS, and PCS also showed substantial improvements compared to baseline (p = 0.005), although the improvement in WOMAC Stiffness was not statistically significant (p = 0.082). Group C (combined therapy) demonstrated the most significant improvements across all parameters, with flexion ROM, WOMAC Pain, WOMAC ADLs, WOMAC Stiffness, and PCS all showing significant changes (p = 0.000). When comparing Group C to Groups A and B, statistically significant differences were found across all metrics (p = 0.000), indicating that Group C experienced the most significant overall improvements (Table 2).

At baseline, no significant differences were observed between groups in ESR, CRP, and IL-6 levels. However, by the 24th session, Group A showed a significant reduction in ESR (from 91 ± 20.9 to 59 ± 6.1, p = 0.002), CRP (from 10.1 ± 7 to 5 ± 3.4, p = 0.002), and IL-6 (from 137 ± 35 to 104 ± 12, p = 0.007). Group B also showed reductions in ESR (from 91 ± 20.9 to 79 ± 6.1, p = 0.005), CRP (from 10.1 ± 7 to 8 ± 3.4, p = 0.006), and IL-6 (from 138 ± 36 to 84 ± 12, p = 0.005). Group C demonstrated the most pronounced reductions, with ESR decreasing from 92 ± 22.9 to 1.1 ± 0.7, CRP from 11.2 ± 6.1 to 6.31 ± 3.0, and IL-6 from 141 ± 38 to 64 ± 12, with statistically significant differences between the groups in all parameters (p < 0.001), indicating superior improvement in Group C (Table 3).

Significant improvements in calcium, magnesium, and phosphorus levels were observed across all groups by the 24th session. In Group A, calcium levels increased from 3.63 ± 0.04 mg/dL to 5.43 ± 0.05 mg/dL (p = 0.007), in Group B from 3.43 ± 0.01 mg/dL to 7.68 ± 0.01 mg/dL (p = 0.005), and in Group C from 3.63 ± 0.04 mg/dL to 9.43 ± 0.05 mg/dL (p = 0.001). Magnesium levels also improved significantly, with Group A rising from 1.8 ± 0.21 mg/dL to 2.00 ± 0.02 mg/dL (p = 0.007), Group B from 1.9 ± 0.22 mg/dL to 2.10 ± 0.04 mg/dL (p = 0.005), and Group C from 1.7 ± 0.01 mg/dL to 2.26 ± 0.02 mg/dL (p = 0.002). Similarly, phosphorus levels increased in Group A from 3.05 ± 0.13 mg/dL to 3.95 ± 0.03 mg/dL (p = 0.005), in Group B from 3.38 ± 0.12 mg/dL to 4.01 ± 0.52 mg/dL (p = 0.003), and in Group C from 3.48 ± 0.32 mg/dL to 4.02 ± 0.52 mg/dL (p = 0.002). Between-group comparisons revealed significant differences across all parameters (p < 0.005), with the most significant improvement observed in Group C (Table 4).

**Figure 2:**
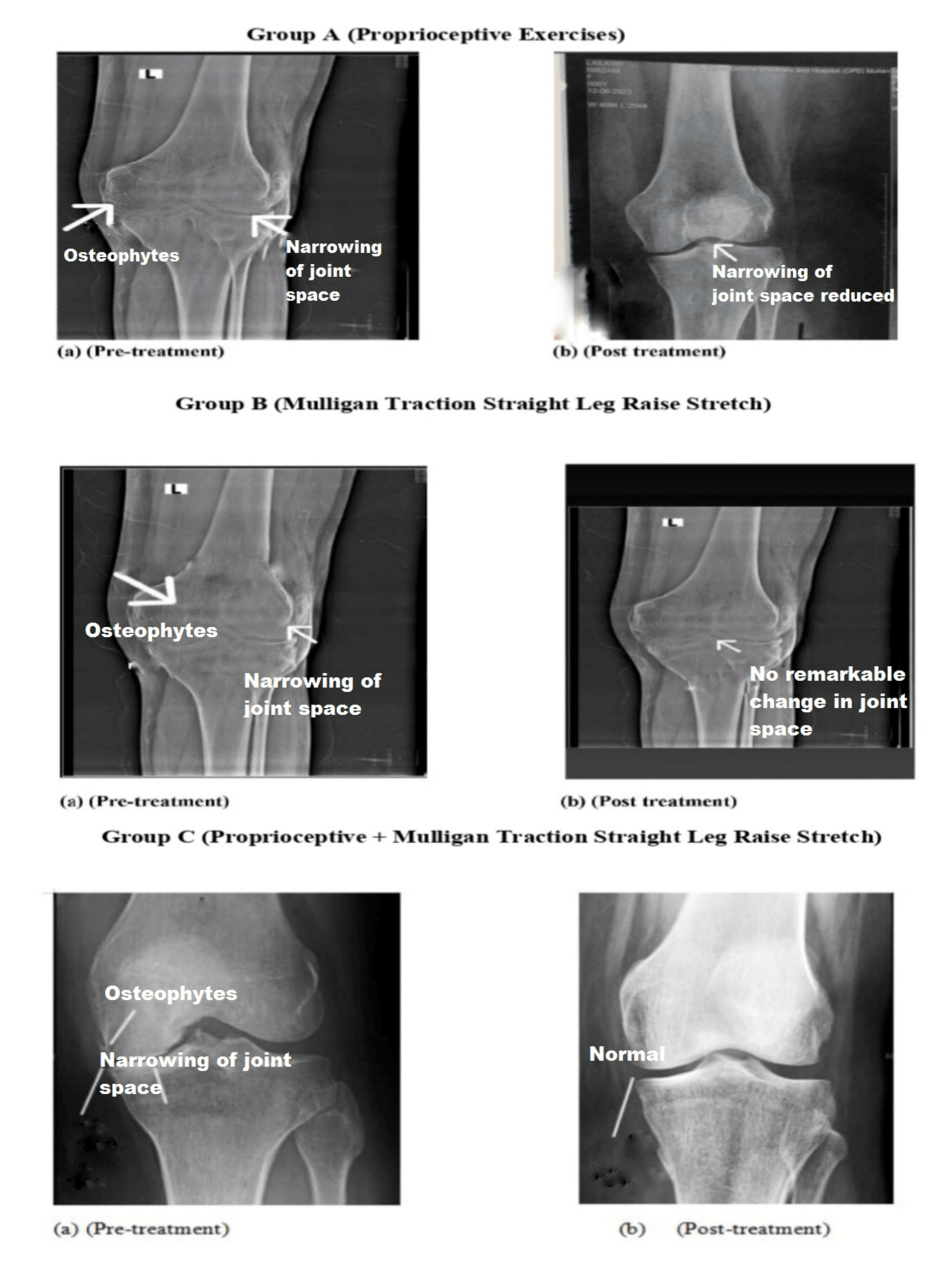
Pictorial representation of X-ray images of osteoarthritic knee joints before and after treatment in the three groups The pre-treatment X-ray of Group A shows joint space narrowing and the presence of osteophytes, indicating joint deterioration. Post-treatment, the joint space appears normalized, and osteophytes are no longer visible, suggesting improvement. Group B’s pre-treatment X-ray also reveals osteophytes and joint space narrowing. However, the post-treatment X-ray shows persistent osteophytes and joint narrowing, indicating minimal or no effect from the treatment. Group C’s pre-treatment X-ray similarly displays osteophytes and joint space narrowing, but the post-treatment X-ray shows a significant improvement, with no visible osteophytes and normalized joint space, indicating a highly significant treatment effect.

X-ray evaluations before and after treatment showed noticeable improvements in Group C, with the attainment of standard joint space compared to Group A. In contrast, Group B showed no significant changes in X-ray results after treatment (Figure 2).

## Discussion

This study assessed the effectiveness of MT-SLR stretch and proprioceptive exercises in patients with knee OA. The investigation examined the effects of proprioceptive exercises, MT-SLR, and combined therapy on clinical and biochemical parameters in OA patients. At baseline, no significant differences were found among the groups regarding ESR, CRP, IL-6, calcium, magnesium, and phosphorus levels. By the 24th session, all interventions led to improvements in these parameters, with Group C (combined treatment) showing the most significant changes. Group A (proprioceptive exercises) exhibited substantial reductions in ESR, CRP, and IL-6, as well as increases in calcium, magnesium, and phosphorus levels. Similarly, Group B (MT-SLR) demonstrated significant reductions in inflammation markers and improvements in mineral levels. However, Group C, which received both treatments, showed the most pronounced improvements in all measured parameters (Table 2, Table 3, Table 4).

These results suggest that combining proprioceptive exercises with MT-SLR offers superior benefits, likely due to the synergistic effects of integrating multiple therapeutic approaches [6]. This supports the concept that multimodal treatments can optimize outcomes more effectively than single interventions alone [6]. A number of biological processes related to inflammation, pain, and mineral metabolism contribute to understanding the molecular mechanisms underlying the effects of proprioceptive exercises, MT-SLR, and their combination in OA [5-7].

Proprioceptive exercises and MT-SLR contribute to reducing inflammation through several molecular mechanisms. Increased joint stability and function from proprioceptive exercises may reduce mechanical stress on the joints, thereby decreasing the release of pro-inflammatory cytokines such as CRP and IL-6 [18]. These exercises improve proprioceptive feedback, enhancing joint alignment and alleviating stress on inflammatory tissues. The transcription factor NF-kB plays a crucial role in inflammatory responses, and its activation is reduced when mechanical stress is lessened, leading to a decrease in the expression of inflammatory cytokines and markers [19].

MT-SLR, a manual traction technique, aids in reducing joint compression and enhancing joint mobility [6]. This mechanical intervention lowers synovial fluid pressure, improving nutrient transport to the joint cartilage and reducing the levels of inflammatory mediators. Additionally, MT-SLR can suppress the activation of matrix metalloproteinases, enzymes that break down cartilage and contribute to inflammation, while also influencing the production of anti-inflammatory mediators [20].

The combined therapy impacts three minerals essential for bone health and inflammation management - calcium, magnesium, and phosphorus. Proprioceptive training promotes bone remodeling by enhancing bone mineral density through increased osteoblastic activity [21], facilitated by signaling pathways like Wnt/β-catenin. MT-SLR exercises induce mechanical loading on the bones, promoting calcium and phosphorus deposition into the bone matrix, thus supporting bone strength [22].

The combination of proprioceptive exercises and MT-SLR may have a synergistic effect, as both strategies work together to improve joint function and reduce inflammation more effectively than either treatment alone.

A limitation of this study is its single-center design and short duration, which prevented the observation of long-term effects. Further research is needed to establish a long-term treatment protocol and evaluate the outcomes of extended treatment periods.

## Conclusions

In the treatment of knee OA, proprioceptive exercises have proven more effective than the MT-SLR stretch. Given the increasing prevalence and severity of knee OA, this study emphasizes the importance of refining and exploring new treatment strategies. The findings clearly indicate that combining MT-SLR with proprioceptive exercises yields superior results compared to either intervention alone. The group receiving the combined therapy showed the most significant improvements in functional mobility, pain reduction, and relevant serological markers, highlighting the potential of this integrated approach to enhance patient outcomes. The notable differences in performance between the treatment groups suggest that a more comprehensive, integrative approach could offer better results in managing knee OA.

## References

[1] Geng R, Li J, Yu C (2023). Knee osteoarthritis: current status and research progress in treatment (review). Exp Ther Med.

[2] Huang L, Zhang Y, Li Q (2024). Investigating the causal relationship between physical activity and incident knee osteoarthritis: a two-sample Mendelian randomization study. Sci Rep.

[3] De Roover A, Escribano-Núñez A, Monteagudo S, Lories R (2023). Fundamentals of osteoarthritis: inflammatory mediators in osteoarthritis. Osteoarthritis Cartilage.

[4] Molnar V, Matišić V, Kodvanj I (2021). Cytokines and chemokines involved in osteoarthritis pathogenesis. Int J Mol Sci.

[5] King LK, Mahmoudian A, Waugh EJ (2023). “You don't put it down to arthritis”: a qualitative study of the first symptoms recalled by individuals with knee osteoarthritis. Osteoarthr Cartil Open.

[6] Ali Z, Zerish Z, Hussain MN (2023). Comparative effects of mulligan traction straight leg raise versus muscle energy technique on pain intensity and hamstring tightness in patient with knee osteoarthritis. Int J Health Sci.

[7] Bhosle P, Rizvi S (2022). Comparison of Mulligan bent leg raise (BLR) versus traction straight leg raise (TSLR) technique in hamstring tightness in sewing machine operators with low back pain. Int J Sci Healthc Res.

[8] Chen J, Chen S (2023). Relationship between mechanoreceptors in the posterior cruciate ligament and patient age or osteoarthritis severity. Orthop J Sports Med.

[9] Wang Y, Wu Z, Chen Z (2021). Proprioceptive training for knee osteoarthritis: a systematic review and meta-analysis of randomized controlled trials. Front Med (Lausanne).

[10] Kurihara C, Kerpel-Fronius S, Becker S (2024). Declaration of Helsinki: ethical norm in pursuit of common global goals. Front Med (Lausanne).

[11] Kang H (2021). Sample size determination and power analysis using the G*Power software. J Educ Eval Health Prof.

[12] Singh AP, Saran S, Thukral BB, Kaushik R (2021). Ultrasonographic evaluation of osteoarthritis-affected knee joints: Comparison with Kellgren-Lawrence grading and pain scores. J Med Ultrasound.

[13] Arif H, Arif N, Kanwal N (2022). Screening of therapeutic potentials of proprioceptive exercises and topical glucosamine sulfate on pain and functional disability in knee osteoarthritis. TJPR.

[14] Sailor S, Limbani A, Dhola D (2020). Association between hamstring flexibility and functional performance of patients with knee osteoarthritis. J Integr Health Sci.

[15] Jo H, Kim K, Im SC (2022). Study of the reliability and validity of the WOMAC Index in patients with total knee replacement. J Korean Soc Phys Med.

[16] Ong WJ, Kwan YH, Lim ZY (2021). Measurement properties of Pain Catastrophizing Scale in patients with knee osteoarthritis. Clin Rheumatol.

[17] Yao M, Xu BP, Li ZJ (2020). A comparison between the low back pain scales for patients with lumbar disc herniation: validity, reliability, and responsiveness. Health Qual Life Outcomes.

[18] Atchison JW, Tolchin RB, Ross BS, Eubanks JE (2020). Manipulation, traction, and massage. Braddom's Physical Medicine and Rehabilitation (Sixth Edition).

[19] Nambi G, Abdelbasset WK, Elsayed SH, Khalil MA, Alrawaili SM, Alsubaie SF (2020). Comparative effects of virtual reality training and sensory motor training on bone morphogenic proteins and inflammatory biomarkers in post-traumatic osteoarthritis. Sci Rep.

[20] de Sire A, Marotta N, Marinaro C, Curci C, Invernizzi M, Ammendolia A (2021). Role of physical exercise and nutraceuticals in modulating molecular pathways of osteoarthritis. Int J Mol Sci.

[21] Erhan B, Ataker Y (2020). Rehabilitation of patients with osteoporotic fractures. J Clin Densitom.

[22] Ward K, Thain PK, Bate G, Woodward M (2024). Therapeutic modalities in sports and exercise therapy. Routledge Handbook of Sports and Exercise Therapy (Second Edition).

